# Biomarkers of Probiotic Therapy in Ulcerative Colitis: A Systematic Review of Mechanisms Underlying Remission

**DOI:** 10.7759/cureus.96126

**Published:** 2025-11-05

**Authors:** Mohammad E Farhad, Ibrahim Belal, Mohamed Baana, Caroline Childs, Moussa Al-Rufayie

**Affiliations:** 1 General Surgery, Hillingdon Hospital, London, GBR; 2 General Medicine, Royal London Hospital, London, GBR; 3 Emergency Medicine, Hillingdon Hospital, London, GBR; 4 Human Development and Health, University of Southampton, London, GBR; 5 Emergency Medicine, London North West University Healthcare NHS Trust, London, GBR

**Keywords:** ibd, inflammation, inflammatory bowel disease, probiotic, probiotics and microbiome, uc, ulcerative colitis

## Abstract

Ulcerative colitis (UC) is a chronic immune-mediated inflammatory bowel disease of unknown aetiology that affects the colon. Patients with UC typically exhibit dysbiosis - an altered gut microbiota profile compared with healthy individuals, who have a more diverse microbial community - suggesting that dysbiosis may be either a cause or a consequence of UC. Current therapeutics include aminosalicylates and steroids with anti-inflammatory and immunosuppressive properties; however, these treatments do not address dysbiosis and instead relieve symptoms. Antibiotics are sometimes used to exert selective pressure on the microbiota and eliminate Gram-negative bacteria, but they are associated with significant side effects, including diarrhoea, *Clostridioides difficile* infection, thrush, and peripheral neuropathy. Probiotics, live microorganisms that confer a health benefit on the host, represent a growing area of interest in the treatment of inflammatory bowel disease (IBD), with the potential to modulate the gut microbiota. Randomised controlled trials (RCTs) measuring biomarkers in patients with UC treated with probiotics were identified through searches of four databases. After screening against predefined inclusion and exclusion criteria, eligible studies were assessed for risk of bias. Thirteen RCTs were included, using interventions such as VSL#3 (Actial Nutrition Inc., Covington, Louisiana) and bifidobacteria-fermented milk (BFM), and measured biomarkers including cytokines over follow-up periods of up to one year. Pro-inflammatory signalling markers, such as nuclear factor-κB (NF-κB), were measured in two of 13 studies and showed significant reductions following probiotic therapy. Conversely, anti-inflammatory biomarkers such as interleukin-10 (IL-10) were significantly increased in all four studies that assessed them. These findings provide insights into the inflammatory pathways targeted by probiotics, including down-regulation of Toll-like receptor 2 (TLR2), leading to reduced pro-inflammatory cytokine production. Such results may inform the development of novel therapeutics targeting these pathways and support the use of biomarkers as objective indicators of disease activity or treatment response, in contrast to potentially biased clinical scores.

## Introduction and background

Introduction

There are more bacterial cells in the human body than human cells [1]. They are distributed across the body, particularly on the skin and in the gastrointestinal tract. Over millions of years, humans have evolved a symbiotic relationship with these microbes. In the gut, most bacteria reside in the large intestine, particularly in the caecum [2]. The collective community of bacteria, viruses, and other microorganisms is termed the gut microbiota, with bacteria as the dominant group. It is estimated that approximately 100 trillion microorganisms inhabit the gut [3]. The four major bacterial phyla present are Firmicutes, Bacteroidetes, Actinobacteria, and Proteobacteria [4]. 

Colonisation begins at birth, when a neonate is exposed to maternal microbes during passage through the birth canal. The microbiota continues to diversify as the host develops [5]. The gut microbiota plays key roles in immunological development, inhibition of pathogenic colonisation, and metabolic functions such as fermentation of dietary fibres [6]. Furthermore, there is evidence linking the gut microbiota to mood and mental health [7]. 

Diet strongly influences microbial diversity. Diets rich in probiotics, prebiotics, and fermented foods such as kefir are associated with increased microbial diversity [4]. A healthy microbiota is also essential for the synthesis of vitamins, including vitamin K and B-group vitamins such as thiamine and riboflavin, which are critical for immune system pathways. The microbiota contributes to carbohydrate metabolism [8]. Dysbiosis and intestinal inflammation may arise from environmental stressors such as pollutants, toxins, diet, sleep disturbance, and psychological stress, reducing beneficial taxa such as Lachnospiraceae and Lactobacillus [9,10]. 

The gut microbiota is a rapidly expanding area of research, with growing evidence linking it to diverse diseases. Promising approaches to modulating the microbiota include faecal microbiota transplantation, probiotics, and prebiotics, particularly in conditions characterised by intestinal inflammation.

Ulcerative colitis (UC) affects approximately one in 420 individuals in the United Kingdom. Although it can present at any age, the most common age of onset is 15-25 years, and it is more prevalent among people of European descent [11]. 

UC is an autoimmune condition in which the immune system mounts an inappropriate response against commensal gut bacteria [11], resulting in recurrent inflammation of the colon. Animal models highlight the importance of the microbiota: genetically engineered mice lacking interleukin-2 (IL-2), interleukin-10 (IL-10), or human leucocyte antigen-B27 (HLA-B27) do not develop colitis under sterile conditions, but do so when colonised with a gut microbiota [12]. A wide range of factors are implicated in UC pathogenesis, including genetic predisposition and exposure to bacterial or viral pathogens. Complications include colonic perforation, severe dehydration, and gastrointestinal bleeding. In advanced disease, primary sclerosing cholangitis and an increased risk of colorectal cancer may also occur [11]. 

The goal of ulcerative colitis treatment is to achieve and maintain remission, thereby preventing relapses and alleviating symptoms. According to the Selecting Therapeutic Targets in Inflammatory Bowel Disease (STRIDE II) consensus, treatment targets in UC should focus on clinical remission, defined as the resolution of rectal bleeding and normalisation of bowel habits, as well as endoscopic remission, characterised by a Mayo endoscopic subscore of 0-1. Therapy selection depends on disease severity. First-line treatments include aminosalicylates and corticosteroids, which exert anti-inflammatory or immunosuppressive effects. Aminoglycoside antibiotics may be used to reduce virulent *Escherichia coli* and other Gram-negative bacteria that carry lipopolysaccharides in their outer membranes. However, while antibiotics can modify the gut microbiota, their use is restricted by adverse effects such as Clostridioides difficile infection, oral thrush, and peripheral neuropathy [13]. Consequently, alternative microbiota-targeted therapies, such as probiotics, have gained increasing attention.

What are Probiotics? 

Probiotics are live microorganisms that, when administered in adequate amounts, confer a health benefit on the host [14]. They occur naturally in fermented foods such as yoghurt [14]. The most commonly studied probiotic strains belong to the genera Bifidobacterium and Lactobacillus. Probiotics modulate gut microbiota, interact with host epithelial and immune systems, and have been used to manage various gastrointestinal disorders, including cases of antibiotic resistance [15]. 

Clinical studies have demonstrated that probiotics can induce remission in UC. For example, Shen et al. reported that VSL#3 (Actial Nutrition Inc., Covington, Louisiana) achieved remission rates comparable to those of 5-aminosalicylic acid, but without adverse effects such as headache, nausea, abdominal pain, or nephrotoxicity [16].

Aim

This systematic review aims to examine RCTs investigating probiotic interventions in patients with UC, with a focus on identifying inflammatory biomarkers responsive to treatment. Identifying biomarkers associated with probiotic therapy may provide new insights into the mechanisms underlying remission, clarify why probiotics improve outcomes, and determine which biomarkers can serve as objective indicators of disease severity-potentially more reliable than clinical scores, which are prone to bias. 

## Review

Methods

A systematic literature search was conducted by one author across four major databases: MEDLINE, Embase, the Cochrane Library, and Web of Science. Database-specific controlled vocabulary (e.g., Medical Subject Headings (MeSH) in MEDLINE and Emtree in Embase) was combined with free-text terms relating to probiotics, ulcerative colitis, and inflammatory biomarkers. Boolean operators and truncation symbols were applied to maximise sensitivity, and search limits were applied where relevant (humans, adults, English language, and randomised controlled trials). The complete search strategies for each database, along with applied restrictions and the number of retrieved records, are summarised in Table 1.

**Table 1:** Search strategy “exp” denotes exploded subject headings; * indicates truncation. Search strategies were adapted to each database. Numbers shown are results before duplicate removal. UC - ulcerative colitis; RCT - randomised controlled trial

Database	Search strategy	Limitations	Results
MEDLINE	(exp Probiotic/ OR Probiotic* OR exp Lactobacillus/ OR Lactobacil* OR exp Bifidobacterium/ OR Bifidobacter* OR “Lactic Acid Bacteria” OR VSL#3 OR Microorgan* OR Bacteri* OR exp Bacteria) AND (exp Gastroenteritis/ OR exp Colitis, Ulcerative/ OR “Ulcerative Colitis” OR Colitis OR UC) AND (exp Inflammation/ OR Inflamm*)	Humans; adults; English language; RCTs	163
Embase	(exp Probiotic agent/ OR Probiotic* OR exp Lactobacillus/ OR Lactobacil* OR exp Bifidobacterium/ OR Bifidobacter* OR “Lactic Acid Bacteria” OR VSL#3 OR Microorgan* OR Bacteri* OR exp Bacteria) AND (Ulcerative Colitis/ OR “Ulcerative Colitis” OR Colitis OR UC) AND (exp Inflammation/ OR Inflamm*)	Humans; adults; English language; RCTs; Embase only	225
Cochrane Library	(Probiotics OR Probiotic* OR Lactobacillus OR Lactobacil* OR Bifidobacterium OR Bifidobacter* OR “Lactic Acid Bacteria” OR VSL#3 OR Microorgan* OR Bacteri*) AND (Gastrointestinal Diseases OR “Ulcerative Colitis” OR Colitis OR Colitis, Ulcerative OR UC) AND (Inflammation OR Inflamm*) AND (Patient* OR Participants OR Volunteers OR Human) AND (Clinical OR Placebo OR “Randomised Controlled Trial” OR “Randomized Controlled Trial”)	Cochrane Gut Trials Group; humans only	25
Web of Science	(Probiotic* OR Lactobacil* OR Bifidobacter* OR “Lactic Acid Bacteria” OR VSL#3 OR Microorgan* OR Bacteri*) AND (“Ulcerative Colitis” OR Colitis OR UC) AND (Inflamm*) AND (Patient* OR Participant* OR Volunteer* OR Human) AND (Clinical OR Placebo OR “Randomised Controlled Trial” OR “Randomized Controlled Trial”)	English language; document type: article	907

Eligibility criteria were defined a priori in accordance with the research objectives. Studies were included if they investigated probiotic interventions in adults with ulcerative colitis and assessed inflammatory biomarkers as outcomes. Only randomised controlled trials (RCTs) were eligible. Exclusion criteria comprised studies involving children, non-UC gastrointestinal disorders, non-probiotic interventions, or studies that reported only clinical or endoscopic outcomes. Additional restrictions were applied with respect to study design, language, and species. A detailed summary of the inclusion and exclusion criteria is presented in Table 2.

**Table 2:** Inclusion and exclusion criteria UC - ulcerative colitis; RCT - randomised controlled trial; CRP - C-reactive protein

Category	Inclusion criteria	Exclusion criteria
Population	Adults (≥18 years); patients with ulcerative colitis	Children (<18 years); Crohn’s disease; other gastrointestinal conditions (e.g., pouchitis, microscopic colitis, irritable bowel syndrome); small bowel disease
Intervention	Probiotics	Prebiotics; antibiotics; synbiotics; faecal microbiota transplantation
Outcomes	Inflammatory biomarkers (e.g., cytokines, CRP, calprotectin)	Clinical outcomes; endoscopic findings
Study design	Randomised controlled trials (RCTs); pilot RCTs	Systematic reviews; meta-analyses; case reports; observational studies; laboratory/in vitro studies; retrospective studies; non-randomised preliminary studies
Date Restriction	No restriction	—
Language	English-language publications	Non-English language
Species	Human studies	Animal studies

Search results were imported into EndNote for duplicate removal and subsequently screened in Rayyan. Title, abstract, and full-text screening were conducted in accordance with the predefined inclusion and exclusion criteria. Following this process, 13 randomised controlled trials were deemed eligible for inclusion in the review. The list of included studies with full reference details is presented in Table 3.

**Table 3:** Articles used in this review This table summarises the 13 randomised controlled trials (RCTs) included in the review. Full citations are presented in Vancouver format. Reference numbers [17–29] correspond to the order of appearance in the main reference list.

Author	Reference
Ballini et al. [17]	Ballini A, Santacroce L, Cantore S, Bottalico L, Dipalma G, Topi S, et al. Probiotics efficacy on oxidative stress values in inflammatory bowel disease: a randomized double-blind placebo-controlled pilot study. Endocr Metab Immune Disord Drug Targets. 2019;19(3):373-381.
Bjarnason et al. [18]	Bjarnason I, Sisson G, Hayee B. A randomised, double-blind, placebo-controlled trial of a multi-strain probiotic in patients with asymptomatic ulcerative colitis and Crohn’s disease. Inflammopharmacology. 2019;27(3):465-473.
Fan et al. [19]	Fan H, Du J, Liu X, Zheng W, Zhuang Z, Wang C, et al. Effects of pentasa-combined probiotics on the microflora structure and prognosis of patients with inflammatory bowel disease. Turk J Gastroenterol. 2019;30(8):680-685.
Hegazy [20]	Hegazy SK. Effect of probiotics on pro-inflammatory cytokines and NF-κB activation in ulcerative colitis. World J Gastroenterol. 2010;16(33):4145-4151.
Huang et al. [21]	Huang M, Chen Z, Lang C, Chen J, Yang B, Xue L, et al. Efficacy of mesalazine in combination with bifid triple viable capsules on ulcerative colitis and the resultant effect on inflammatory factors. Pak J Pharm Sci. 2018;31(6):2891-2895.
Ishikawa et al. [22]	Ishikawa H, Akedo I, Umesaki Y, Tanaka R, Imaoka A, Otani T. Randomized controlled trial of the effect of bifidobacteria-fermented milk on ulcerative colitis. J Am Coll Nutr. 2003;22(1):56-63.
Kato et al. [23]	Kato K, Mizuno S, Umesaki Y, Ishii Y, Sugitani M, Imaoka A, et al. Randomized placebo-controlled trial assessing the effect of bifidobacteria-fermented milk on active ulcerative colitis. Aliment Pharmacol Ther. 2004;20(10):1133-1141.
Kruis et al. [24]	Kruis W, Fric P, Pokrotnieks J, Lukas M, Fixa B, Kaščák M, et al. Maintaining remission of ulcerative colitis with the probiotic Escherichia coli Nissle 1917 is as effective as with standard mesalazine. Gut. 2004;53(11):1617-1623.
Li et al. [25]	Li G, Zeng S, Liao W, Lv N. The effect of bifid triple viable on immune function of patients with ulcerative colitis. Gastroenterol Res Pract. 2012;2012:418349.
Wildt et al. [26]	Wildt S, Nordgaard I, Hansen U, Brockmann E, Rumessen JJ. A randomised double-blind placebo-controlled trial with Lactobacillus acidophilus La-5 and Bifidobacterium animalis subsp. lactis BB-12 for maintenance of remission in ulcerative colitis. J Crohns Colitis. 2011;5(2):115-121.
Ng et al. [27]	Ng SC, Plamondon S, Kamm MA, Hart AL, Al-Hassi HO, Guenther T, et al. Immunosuppressive effects via human intestinal dendritic cells of probiotic bacteria and steroids in the treatment of acute ulcerative colitis. Inflamm Bowel Dis. 2010;16(8):1286-1298.
Yilmaz et al. [28]	Yilmaz I, Dolar M, Ozpinar H. Effect of administering kefir on the changes in fecal microbiota and symptoms of inflammatory bowel disease: a randomized controlled trial. Turk J Gastroenterol. 2019;30(3):242-253.
Cui et al. [29]	Cui HH, Chen CL, Wang JD, Yang YJ, Cun Y, Wu JB, et al. Effects of probiotic on intestinal mucosa of patients with ulcerative colitis. World J Gastroenterol. 2004;10(10):1521-1525.

We assessed the quality of the included trials using the Cochrane risk of bias tool (RoB 1) for RCTs. Each study was judged across domains such as randomisation, allocation concealment, blinding, reporting, and completeness of outcome data. Overall risk of bias was then categorised for each trial. A summary is shown in Table 4, with the full detailed assessment provided in Appendix Table 10.

**Table 4:** Risk of Bias Assessment of Included Studies (Cochrane Tool) Risk of bias was assessed using the Cochrane tool (RoB 1) across seven domains. Overall risk was classified as Good, Fair, or Poor based on domain-level judgements.

Study	Random sequence generation	Allocation concealment	Selective reporting	Other sources of bias	Blinding (participants and personnel)	Blinding (outcome assessment)	Incomplete outcome data	Overall risk of bias
Ballini et al. [17]	Unclear	Low	Unclear	Low	Low	Unclear	Low	Fair
Bjarnason et al. [18]	Low	Low	Unclear	Low	Low	Low	Low	Fair
Fan et al. [19]	Low	Unclear	Unclear	Low	High	Unclear	Low	Fair
Hegazy [20]	Low	Unclear	Unclear	Low	Unclear	Unclear	High	Poor
Huang et al. [21]	Low	Low	Low	Low	Low	Low	Low	Good
Ishikawa et al. [22]	Low	Low	Low	Low	High	Unclear	Low	Fair
Kato et al. [23]	Low	Low	Low	Low	Low	Low	Low	Good
Kruis et al. [24]	Low	Low	Low	Low	Low	Low	Low	Good
Li et al. [25]	Low	Low	Low	Low	Unclear	Low	Low	Fair
Wildt et al. [26]	Low	High	Low	Low	Low	Low	Low	Fair
Ng et al. [27]	Low	Unclear	Low	Low	Low	Unclear	Low	Fair
Yilmaz et al. [28]	Low	High	Low	Low	High	High	Low	Poor
Cui et al. [29]	Unclear	Unclear	Low	Low	Low	Unclear	Low	Fair

Results

**Figure 1:**
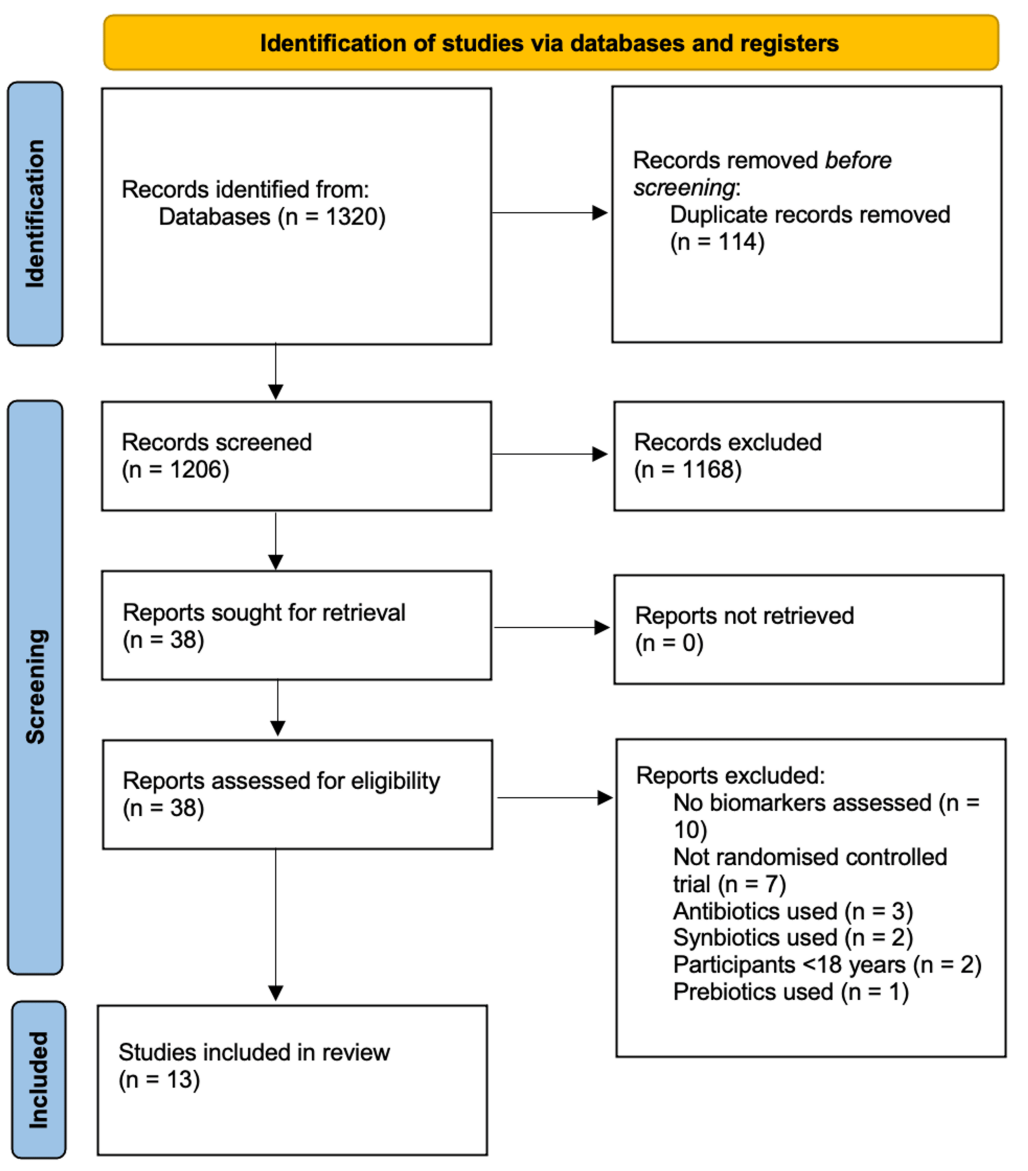
PRISMA flow diagram of the study selection process PRISMA - Preferred Reporting Items for Systematic Reviews and Meta-Analyses

Figure 1 presents a Preferred Reporting Items for Systematic Reviews and Meta-Analyses (PRISMA) flow diagram outlining the systematic study selection process. Initially, 1320 records were identified through database searches, and 114 duplicates were removed, leaving 1206 records for screening. After title and abstract screening, 1168 records were excluded, and 38 full-text articles were retrieved for assessment. Following full-text review, 25 reports were excluded (10 did not assess biomarkers, seven were not randomised controlled trials, three used antibiotics, two used synbiotics, and one used prebiotics), resulting in 13 studies meeting all inclusion criteria for the final review. See Appendix Table 11 for the results of the included studies.

Most of these studies measured a variety of biomarkers. Although some did not use biomarkers as their primary objective, they still measured them because they showed strong correlations. Furthermore, different biomarkers were measured using different methods and different probiotics, yet they showed similar results. These are presented in the tables below. 

C-Reactive Protein 

Studies that measured C-reactive protein are outlined in Table 5, which also indicates whether there were significant differences in post-treatment findings. 

**Table 5:** Studies that measured C-reactive protein, findings after probiotic treatment

Study	Findings post treatment
Bjarnason et al. [18]	No significant difference (p>0.05)
Fan et al. [19]	Significantly lower in observation group than control group (p<0.05)
Kruis et al. [24]	Data not provided
Wildt et al. [26]	No significant difference (data not provided)
Yilmaz et al. [28]	No significant difference

Five studies measured C-reactive protein (CRP). All except Fan et al. showed no significant difference in CRP levels compared with the control group. CRP is produced by the liver in response to inflammation and is widely used as an indicator of inflammatory activity [30]. In ulcerative colitis, CRP does not consistently change following probiotic therapy. Participants in Fan et al.’s study had CRP >60 mg/L before probiotic treatment [19]. Apart from Fan et al.’s study, the other studies showed no significant difference in CRP. This discrepancy may relate to the probiotic used: Fan et al. used Bifico, which contains different strains of Lactobacillus and Bifidobacterium than those used by the other authors [19]. Probiotic formulation may influence whether CRP falls. Three of the studies that measured CRP also administered mesalazine (5-aminosalicylic acid) to both groups; this anti-inflammatory medication can affect CRP levels [31]. 

Cytokines 

Several of the included trials evaluated changes in cytokine expression as biomarkers of inflammation in ulcerative colitis. These studies assessed both pro-inflammatory and anti-inflammatory cytokines using a variety of laboratory techniques, including enzyme-linked immunosorbent assays (ELISA), immunohistochemistry, and reverse transcriptase polymerase chain reaction (RT-PCR). The specific cytokines measured, the methods employed, and the main post-treatment findings for each study are summarised in Table 6.

**Table 6:** Studies that measured cytokines, methods used, and findings after probiotic treatment ELISA - enzyme-linked immunosorbent assay; mRNA - messenger ribonucleic acid

Study	Cytokines measured	Method used	Findings post treatment
Fan et al. [19]	Interleukin-6; Interleukin-4	Enzyme-linked immunosorbent assay – measured serum	Interleukin-6 was significantly lower in the observational group compared to the control group (p<0.05). Interleukin-4 was significantly higher in the observational group compared to the control group (p<0.05).
Huang et al. [21]	Tumour necrosis factor alpha; Interleukin-8; Interleukin-10	Enzyme-linked immunosorbent assay – measured serum	Tumour necrosis factor alpha and Interleukin-8 showed a significant decrease in control and probiotic groups. Interleukin-10 increased significantly (p<0.01).
Li et al. [25]	Interleukin-1 beta; Interleukin-10	Immunohistochemistry according to the procedure of the SP9000 kit – measured colon mucosa	Interleukin-10 increased significantly (p<0.01). Interleukin-1 beta decreased significantly.
Hegazy [20]	Interleukin-6; Tumour necrosis factor alpha	Interleukin-6 – enzyme-linked immunosorbent assay measured using supernatant from colon tissue. Tumour necrosis factor alpha – detection of messenger ribonucleic acid expression using reverse transcriptase polymerase chain reaction	Interleukin-6 decreased significantly. Tumour necrosis factor alpha messenger ribonucleic acid expression decreased significantly.
Cui et al. [29]	Interleukin-1; Tumour necrosis factor alpha; Interleukin-10	Reverse transcriptase – polymerase chain reaction using colonic biopsy	Interleukin-1 showed no significant change; however, there was a significant increase in the placebo group. Tumour necrosis factor alpha showed a significant decrease (p<0.01) (significant increase in the placebo group, p<0.01). Interleukin-10 showed a significant increase (p<0.01) (significant decrease in the placebo group).
Ng et al. [27]	Interleukin-10; Interleukin-8; Interleukin-6; Interleukin-4; Interleukin-5; Interleukin-1 beta; Tumour necrosis factor alpha; Interleukin-12p70; Interleukin-13	Enzyme-linked immunosorbent assay – used supernatants from colonic biopsy	Significant increase in Interleukin-10. Interleukin-12p70 showed a significant decrease. Dendritic cell activity was significantly greater in ulcerative colitis patients. Interleukin-6 and Interleukin-13 showed no significant difference.

A cytokine is an umbrella term used to describe substances such as interferons, interleukins, and growth factors, which are secreted mainly by cells of the immune system. They have autocrine, paracrine, and endocrine functions; they are vital in cell signalling and act as pro-inflammatory or anti-inflammatory messengers in inflammatory cascades [32].

Cytokines are often viewed as the orchestra of inflammatory responses. They have many functions, often more than one. Cytokines such as Interleukin-1, Interleukin-6, and tumour necrosis factor alpha are pro-inflammatory, whereas cytokines such as Interleukin-10 and Interleukin-11 are anti-inflammatory. Interleukin-4 has context-dependent roles, acting in some cases as pro-inflammatory and in others as regulatory. From the results gathered, it is clear that probiotics have a significant effect on both pro- and anti-inflammatory cytokines. Measuring cytokines is very useful for detecting and quantifying inflammation.

The methods used to measure cytokines varied (Table 6). Some studies examined serum; others used biopsies. Three main tools were used to measure cytokines: enzyme-linked immunosorbent assay, reverse transcription polymerase chain reaction, and immunohistochemistry. All three approaches showed changes in cytokine levels post-treatment with probiotics; there were more similarities than differences between studies.

Six studies measured pro-inflammatory cytokines; a common one was Interleukin-6. Interleukin-6 has diverse functions (pleiotropic activity). In the context of ulcerative colitis, its main role is as a pro-inflammatory messenger, stimulating acute-phase proteins such as C-reactive protein, serum amyloid A, fibrinogen, and hepcidin from hepatocytes. It also inhibits albumin production, allowing increased substances to seep through capillaries into tissue [33]. Two of the three studies measuring Interleukin-6 reported a significant decrease after probiotic treatment. Ng et al.’s study did not, which may relate to its high dropout rate (29%) and that it measured dendritic-cell-derived Interleukin-6 only. Another cytokine significantly affected by probiotics was tumour necrosis factor. This cytokine is mostly secreted by macrophages and has a wide array of functions in the inflammatory cascade, such as proliferation, differentiation, apoptosis, lipid metabolism, and coagulation [34]. Therefore, high levels indicate inflammation. All studies that measured tumour necrosis factor alpha found levels higher than normal. Different methods were used to measure it. Huang et al. used an enzyme-linked immunosorbent assay and found a significant decrease after treatment. Similarly, Hegazy and Cui et al., who both used reverse transcription polymerase chain reaction, found significant decreases post-treatment. By contrast, Ng et al.’s results did not show a significant difference, potentially due to the probiotic used or to measuring dendritic-cell-derived cytokines only. Huang’s control group also showed a decrease, which may reflect mesalazine’s anti-inflammatory properties.

Other cytokines measured included Interleukin-4, Interleukin-18, and Interleukin-8; all showed significant differences post-treatment. Interleukin-4 has many functions, including activating naïve T cells and B cells. Interleukin-18 and Interleukin-8 are mainly produced by macrophages; they are responsible for activating other inflammatory cells such as neutrophils [32]. All studies except Ng et al. found significant decreases in Interleukin-4, Interleukin-18, and Interleukin-8. Huang et al.’s control group also saw a significant decrease in Interleukin-8; the use of mesalazine and the absence of a placebo could explain these results [21]. Li et al. reported no significant difference in cytokines post-treatment [25].

Anti-inflammatory cytokines are responsible for restoring homeostasis through negative feedback after an inflammatory cascade. Unfortunately, in autoimmune diseases such as ulcerative colitis, chronic inflammation does not seem to be controlled by naturally produced anti-inflammatory cytokines. Therefore, it is vital for therapeutics to target these cytokines and increase their levels to help achieve remission. One key cytokine is Interleukin-10. Interleukin-10 plays a vital role in regulating inflammatory cascades; it downregulates T helper type 1 cytokines as well as major histocompatibility complex class II antigen and various stimulatory molecules on macrophages [35,36]. Four of the studies measured Interleukin-10; all found a significant increase post-treatment, regardless of probiotic, dose, or measurement method.

Faecal Biomarkers

Several trials assessed faecal biomarkers as indicators of inflammation and gut microbial activity. These included calprotectin, lactoferrin, and short-chain fatty acids (SCFAs). The biomarkers measured and their post-treatment findings are summarised in Table 7.

**Table 7:** Studies that measured faecal biomarkers and findings after probiotic treatment

Study	Biomarkers measured	Findings post treatment
Bjarnason et al. [18]	Calprotectin	Decreased, not significantly
Fan et al. [19]	Lactoferrin	Significantly lower
Ishikawa et al. [22]	Organic acids: succinate, lactate, formate, acetate, propionate, butyrate, valerate	No significant difference in organic acids, except butyrate. Butyrate decreased significantly (p<0.05).
Kato et al. [23]	Organic acids: succinate, lactic acid, acetic acid, propionic acid, butyric acid, total short-chain fatty acids	Propionic acid increased significantly (p<0.05). Butyric acid increased significantly (p<0.05). Total short-chain fatty acids increased significantly (p<0.05).
Hegazy [20]	Calprotectin	Significant decrease

Faecal biomarkers such as calprotectin are currently among the first-line diagnostic tools for gastrointestinal symptoms related to inflammatory bowel disease; they reflect the condition of the colonic mucosa [36]. Bjarnason et al. found a non-significant decrease in calprotectin following probiotic treatment, whereas Hegazy observed a significant decrease. This difference may relate to the probiotic strain and the longer duration in Hegazy’s study. It may also take time for calprotectin levels to fall following probiotic therapy. Hegazy used sulfasalazine (a disease-modifying anti-rheumatic drug in other contexts), and Bjarnason et al. used 5-aminosalicylic acid and azathioprine; these drugs have immunomodulatory and anti-inflammatory effects that may have influenced results.

Like calprotectin, lactoferrin is a reliable biomarker for intestinal inflammation. It is secreted by inflammatory cells such as neutrophils during inflammation [37,38]. Fan et al.’s study was the only one to measure lactoferrin, showing a significant decrease after probiotic treatment. However, patients were on Pentasa (mesalazine), which has immunomodulatory properties and may have affected these results.

Although organic acids are not strictly inflammatory biomarkers, they can reflect gut activity. Interactions between microflora and the immune system produce organic acids that may differ from a normal gastrointestinal tract and can be detected in faeces during intestinal inflammation [39,40]. Ishikawa et al. found no significant differences in faecal organic acids except for a decrease in butyrate, whereas Kato et al. reported significant increases in butyrate, propionate, and total SCFAs after probiotic treatment. These results provide limited insight into the mechanisms by which probiotics improve remission in ulcerative colitis.

Serum biomarkers 

Studies that measured serum biomarkers are shown in Table 8, including the specific biomarkers assessed and their post-treatment findings. These biomarkers included markers of oxidative stress, inflammatory and immune parameters, and general biochemical measures such as haemoglobin, albumin, and immunoglobulins. While some trials reported significant improvements, many found no notable differences after probiotic treatment.

**Table 8:** Serum biomarkers and findings after probiotic treatment “No significant difference” refers to comparison with control groups. p<0.05 was considered statistically significant. CD - cluster of differentiation; ROS - reactive oxygen species

Study	Biomarkers measured	Findings post treatment
Ballini et al. [17]	Oxidative stress (biological antioxidant potential; derivatives-reactive oxygen metabolites), leucocytosis, vitamin B12	Significant decrease in reactive oxygen species. No other data shown.
Bjarnason et al. [18]	Haemoglobin, haematocrit, white blood cell count, erythrocyte sedimentation rate	No significant difference.
Fan et al. [19]	Alpha-1 antitrypsin; Beta-2 microglobulin	Significantly lower (p<0.05).
Ishikawa et al. [22]	Total protein, albumin, total cholesterol, triglycerides	No significant difference.
Kruis et al. [24]	Erythrocyte sedimentation rate, orosomucoid, blood count, liver enzymes, creatinine, iron, albumin	Data not provided.
Li et al. [25]	Immunoglobulins, complement C3 and C4, T-cell group, erythrocyte sedimentation rate, CD4 T cells, CD8 T cells	No significant difference. Ratio of CD4 T cells to CD8 T cells increased significantly more than control group (p<0.05).
Wildt et al. [26]	Haemoglobin, white blood cell count, albumin	No significant difference.
Yilmaz et al. [28]	Haemoglobin, erythrocyte sedimentation rate	No significant difference.

Eight studies measured blood serological markers; most did not find significant differences post-treatment. 

Fan et al. measured alpha-1 antitrypsin, a multifunctional glycoprotein produced by the liver with anti-inflammatory, immunomodulatory, and tissue-repair-related roles [39]. Fan et al. found a significant decrease in alpha-1 antitrypsin compared to pre-treatment, where there was no significant difference between the test and control groups. They also measured beta-2 microglobulin, an important modulator of lymphocyte surfaces and immune regulation associated with other inflammatory diseases such as rheumatoid arthritis [39]. Fan et al. found a significant decrease in beta-2 microglobulin in the serum after probiotic treatment. T cells are important immune cells in adaptive immunity; Li et al. measured both CD4 and CD8 T cells. CD4 T cells have many functions, including activating B cells to release antibodies and activating CD8 T cells to induce pores using perforin in target cells. Li et al.’s results showed an increased CD4 to CD8 ratio compared to the control group. A high CD4:CD8 T-cell ratio is associated with autoimmune diseases. 

Other Colonic Biomarkers 

Studies that measured other colonic biomarkers are shown in Table 9, including the specific biomarkers assessed and their post-treatment findings. These biomarkers included colonic myeloperoxidase, nuclear factor kappa-light-chain-enhancer of activated B cells (NF-κB p65), dendritic cell markers, and Toll-like receptors (TLRs). Several trials reported significant reductions in inflammatory activity, while others demonstrated selective effects on dendritic cell function and receptor expression.

**Table 9:** Studies that measured other colonic biomarkers NF-κB - nuclear factor kappa-light-chain-enhancer of activated B cells; CD - cluster of differentiation; TLR - Toll-like receptor

Study	Biomarkers measured	Findings post treatment
Hegazy [20]	Colonic myeloperoxidase; Nuclear factor kappa-light-chain-enhancer of activated B cells (p65)	Significantly decreased
Ng et al. [27]	CD11c-positive myeloid dendritic cells (measured by cytokine production). Dendritic cell expression of CD40, CD86, Toll-like receptor-2 (TLR2), Toll-like receptor-4 (TLR4)	Significant increase in Interleukin-10 and decrease in Interleukin-12p40 production by colonic dendritic cells. Toll-like receptor-2 (TLR2) showed a significant decrease. No other significant difference.
Cui et al. [29]	Nuclear factor kappa-light-chain-enhancer of activated B cells; Inhibitor of kappa-light-chain-enhancer of activated B cells	Significant decrease in Nuclear factor kappa-light-chain-enhancer of activated B cells expression (p<0.01). Inhibitor of kappa-light-chain-enhancer of activated B cells showed no significant decrease.

Myeloperoxidase is a peroxidase enzyme abundantly expressed in neutrophil granulocytes. It is a key element of the innate immune system, found primarily in lysosomal azurophilic granules in neutrophils. Hegazy’s results showed a significant decrease in colonic myeloperoxidase, indicating decreased inflammation [20]. 

Both Hegazy and Cui et al. measured nuclear factor kappa-light-chain-enhancer of activated B cells, a pathway linked to prototypical pro-inflammatory signalling due to its role in activating pro-inflammatory genes, including cytokines, chemokines, and adhesion molecules [41]. Both studies showed significant decreases, indicating suppression of these inflammatory pathways. 

Ng et al. measured cytokines produced specifically by colonic dendritic cells, as well as expression molecules. They found a significant increase in the anti-inflammatory cytokine Interleukin-10 and a significant decrease in the pro-inflammatory cytokine Interleukin-12p40, which may indicate decreased dendritic cell activity [42]. Their results also showed a significant decrease in expression of Toll-like receptor 2, which forms heterodimers with Toll-like receptor 1 and Toll-like receptor 6 and represents an initial step of an inflammatory cascade in the innate and adaptive response [43].

Discussion

This systematic review aimed to determine which biomarkers were measured by studies investigating the relationship between ulcerative colitis and probiotics and to identify significant post-treatment changes. This improves understanding of which inflammatory markers orchestrate this disease and how they may be manipulated by probiotics, which have been shown to be safer than antibiotics and steroids due to relatively fewer side effects [15]. Furthermore, identifying markers indicative of disease severity will help determine which bacterial strains are most beneficial for patients with ulcerative colitis, providing a measure more reliable than clinical data. This review has not answered the optimal-strain question because the probiotics used contained different strains; overall, they performed similarly. Generally, certain biomarkers-especially inflammatory markers-showed significant changes after probiotic treatment, and many markers within the same pathway changed concordantly. 

Many studies assessing the relationship between ulcerative colitis and probiotics measure disease severity using clinical scores such as the Ulcerative Colitis Endoscopic Index of Severity (UCEIS). The UCEIS is an endoscopic scoring system based on three components: vascular pattern, bleeding, and presence of erosions or ulcers, each graded from 0 to 3, with a total score ranging from 0 (normal) to 9 (severe disease). This can present problems when comparing participants and studies because there is more room for bias and reliance on observer judgment. Peyrin-Biroulet explains that there is no formal, validated, or consensus definition of mild, moderate, or severe inflammatory bowel disease [32]. Although C-reactive protein levels are used in practice, this does not always reflect disease severity, as seen in studies in this review reporting no change in C-reactive protein while significant changes in other biomarkers were noted. Furthermore, ulcerative colitis is broad in terms of severity and stage. Patients present with different symptoms, and clinical findings are classified into broad subgroups, which complicates tailoring treatment to specific groups [33].

Biomarkers such as inflammatory cytokines provide a more scientific and accurate description of disease severity, which would help clinicians target treatment more appropriately. Identifying affected biomarkers offers insight into inflammatory pathways involved in the aetiology and chronic inflammation of ulcerative colitis. It also helps explain why probiotics improve inflammation and increase remission in these patients. Understanding which markers to monitor will improve the identification of probiotics most effective at reducing colonic inflammation and increasing remission rates. 

The current National Institute for Health and Care Excellence guideline uses faecal calprotectin as one of the first-line diagnostic tools for patients presenting with inflammatory bowel disease-like symptoms; it provides an inexpensive, easy, and rapid alternative to endoscopy [23]. Bjarnason et al. and Hegazy found that the probiotics they used reduced faecal calprotectin significantly. Calprotectin is a reliable biomarker for measuring colonic inflammation [23]. In Bjarnason et al.’s study, four patients experienced clinical relapse; all were on placebo and had calprotectin levels exceeding 250 micrograms per gram. This indicates that calprotectin accurately reflects relapse or remission. However, it does not explain mechanisms because it is produced as a result of intestinal inflammation. Unfortunately, Bjarnason et al. and Hegazy used 5-aminosalicylic acid and sulfasalazine in control groups, which also showed falls in calprotectin. Hegazy also used sulfasalazine in combination with probiotics in the test group. This could indicate that combination treatment may be more effective. 

Cytokines, peptide or glycoprotein messengers secreted from inflammatory cells such as macrophages and neutrophils, regulate the inflammatory process across pathways [19]. In ulcerative colitis, pro-inflammatory cytokines are generally higher than in healthy people [19]. Six papers measured cytokines before and after probiotic treatment; most found significant changes, especially consistent increases in Interleukin-10 across all studies that measured it, regardless of method and probiotic used. Huang found no significant baseline differences between groups but a significant post-treatment increase in Interleukin-10 in the probiotic group. Ng et al. found no detectable Interleukin-10 in healthy patients; among probiotic responders, Interleukin-10 increased significantly, whereas in non-responders the increase was smaller. This may indicate that Interleukin-10 suppresses other cytokines without resolving upstream drivers. Interleukin-10 is produced naturally in ulcerative colitis, indicating that it is a result of inflammation, not a cause. Nevertheless, this supports a mechanism where probiotics increase anti-inflammatory cytokines to suppress pro-inflammatory mediators, improving colon health. Interleukin-10 is also known to block nuclear factor kappa-light-chain-enhancer of activated B cells activity [19]. Interestingly, Hegazy and Cui et al. found a significant decrease in nuclear factor kappa-light-chain-enhancer of activated B cells activity post-treatment with probiotics, possibly due to increased Interleukin-10. The canonical nuclear factor kappa-light-chain-enhancer of activated B cells pathway is defined primarily in response to tumour necrosis factor alpha and Interleukin-1 signalling. Other studies in rheumatoid arthritis show that pro-inflammatory cytokine and chemokine production by diseased tissue is nuclear-factor-kappa-B-dependent. Although this pathway is not fully understood, nuclear factor kappa-light-chain-enhancer of activated B cells activates both innate and adaptive pro-inflammatory cells and cytokines [29]. Therefore, probiotics may influence this pathway directly and indirectly through other cytokines. 

Most studies showed decreases in pro-inflammatory cytokines such as Interleukin-6 (IL-6) and tumour necrosis factor alpha (TNF-α), and in some contexts Interleukin-4 (IL-4), except in Ng et al.’s study, which assessed dendritic-cell-derived cytokines. In Ng et al.’s study, Interleukin-12p70 (IL-12p70) decreased significantly. This cytokine, released mainly from activated dendritic cells, is thought to be a component of bioactive Interleukin-12 and Interleukin-23. Furthermore, in phase 2b clinical trials, anti-IL-12p70 therapy has been effective in Crohn’s disease [30]. Therefore, decreased IL-12p70 may represent another probiotic target. Ng et al. also found that Toll-like receptor 2 expression on dendritic cells decreased. Toll-like receptors are pattern recognition receptors that detect foreign antigens and present them to T cells [27]. Since ulcerative colitis has autoimmune features, Toll-like receptor 2 may present self-antigen to T cells. Results from Ng et al. showed that probiotics caused significant down-regulation of Toll-like receptor 2 [31], indicating another pathway that probiotics can inhibit. 

Other studies, such as Ballini et al., showed that probiotics enhanced antioxidant defences that remove reactive oxygen species. These unstable oxygen-containing molecules readily react with cellular molecules. Although reactive oxygen species are essential during appropriate immune responses to microbial invasion, in ulcerative colitis, there is increased oxidative stress due to reactive oxygen species accumulation. Reactive oxygen species can serve as intra- and intercellular messengers, influencing gene expression and activating signalling cascades, including inflammatory ones [28]. They can also mediate activation of the redox-sensitive transcription factor nuclear factor kappa-light-chain-enhancer of activated B cells [31]. Unfortunately, the colon is prone to damage by reactive oxygen species due to limited access to antioxidants, which probiotics may improve [28]. Patients with ulcerative colitis have significantly higher myeloperoxidase levels than healthy people [28]. From two studies, we see that probiotics have antioxidant activity: Ballini et al. showed significant decreases in derivatives-reactive oxygen metabolites and biological antioxidant potential tests, and Hegazy reported decreased colonic myeloperoxidase. This indicates that colonic susceptibility to reactive oxygen species could contribute to ulcerative colitis and can be targeted using probiotics. 

Inflammation is a complex cascade full of negative and positive feedback systems, pathways, triggers, regulators, and transcription factors. This review has identified key inflammatory mechanisms that the 13 studies were able to manipulate using probiotics. Collectively, the findings demonstrate why probiotics increase remission and improve colonic inflammation in patients with ulcerative colitis. The future of ulcerative-colitis therapy using probiotics appears promising due to their multiple actions on the inflammatory cascade and favourable side-effect profile. 

Strengths of the studies 

All 13 studies were randomised controlled trials. All except one reported random sequence generation; risks in selective reporting and outcome data were generally low. This led to all but two papers scoring fair or good using the Cochrane risk-of-bias tool for randomised controlled trials. Furthermore, seven of the 13 studies used a placebo, helping to provide more accurate and reliable results. All studies had an almost equal ratio of male to female participants and covered a wide adult age range. All but one provided participant demographics. 

Weaknesses of the studies 

Although findings were broadly similar, there were numerous weaknesses. As shown in Table 4, using the Cochrane risk-of-bias tool, only three studies were rated good; the rest were poor or fair (see Appendix for details). Because ulcerative colitis is a chronic inflammatory disease, most participants were receiving maintenance therapy with 5-aminosalicylic acid, azathioprine, salazosulfapyridine/sulfasalazine, and glucocorticoids; Ishikawa et al. even used steroids. All these drugs have immunosuppressive or anti-inflammatory effects. Understandably, it would have been unethical to withhold treatment. This undoubtedly skewed results, evident in Huang et al.’s study, where the mesalazine-treated control group also had a significant increase in Interleukin-10 and a significant decrease in oxidative stress. Similar results were seen in Bjarnason et al.’s study, which found a greater decrease in C-reactive protein in the placebo group using 5-aminosalicylic acid. Some studies, such as Yilmaz et al., did not use a placebo because it was difficult to create a believable kefir placebo. Five of the studies did not use a placebo. 

A recurring theme was that many studies measured clinical scores as their primary objective, leaving biomarkers as secondary. Kruis did not publish biomarker data even though they were measured. Furthermore, demographics were sometimes unclear (e.g., missing sex or age). Although volunteers had ulcerative colitis, patients differed widely in disease severity and duration, complicating comparisons. Probiotics used included varied strains; it would be useful to examine specific strains to determine whether they drive biomarker changes. 

Some studies, such as Hegazy, recruited 40 participants but published data on only six. Several studies were multicentre and multinational, which may have introduced inaccuracies due to miscalibration of equipment and measurement tools. Lastly, dropout rates were high in many studies, especially Ng et al. (eight participants), leading to smaller sample sizes, which was a general issue and may explain discrepant cytokine results. 

Strengths of this review 

The main strength is that these mechanistic questions have not previously been synthesised in humans: which pathways can be manipulated by probiotics to inform strain choice, why they work, and what that implies for causative factors in ulcerative colitis. Most reviews focus on probiotics and clinical data. Some have examined mechanisms and biomarkers, but these are mostly animal models. The objective was clearly stated. Using inclusion and exclusion criteria, four databases were searched to find relevant papers; duplicates were removed, papers were screened, and clear reasons were provided for exclusions. Thirteen papers met all criteria and were assessed for bias and quality using the Cochrane risk-of-bias tool for randomised trials. This review strictly required probiotics (not synbiotics or prebiotics). The search strategy, methods, and included papers are shown clearly in tables. 

Weaknesses of this review 

Although correlations, patterns, and explanations were identified, only 13 studies were included. With more data, stronger conclusions and correlations could be made. This review was conducted by one person; although papers were screened twice, having a second reviewer would reduce screening bias, as some papers with promising data might have merited inclusion. 

## Conclusions

In conclusion, this review has demonstrated that probiotics have anti-inflammatory, immunomodulatory, and antioxidant properties. Probiotics could be the future of inflammatory bowel disease treatment; although alone they may not be sufficient, using them alongside other therapeutics such as 5-aminosalicylic acid may improve remission and reduce colonic inflammation. Probiotics appear to act through multiple mechanisms that are not yet fully understood; however, this review identified clear links and pathways that probiotics may modulate, such as the canonical nuclear factor kappa-light-chain-enhancer of activated B cells pathway, as well as reducing oxidative stress and increasing anti-inflammatory markers such as Interleukin-10. This is reflected in biomarker changes. Therefore, measuring biomarkers and examining their interrelationships enables a better understanding of ulcerative colitis aetiology, the inflammatory pathways involved, and why probiotics improve remission and reduce inflammation. There remains substantial room for research: many animal models examine specific inflammatory markers, but human studies on ulcerative colitis and probiotics should focus more on inflammatory markers rather than clinical scores. Future studies should evaluate individual bacterial strains and identify which are most effective. Effectiveness should be measured by biomarkers such as reductions in pro-inflammatory markers, nuclear factor kappa-light-chain-enhancer of activated B cells activity, oxidative stress, Toll-like receptor expression, and increases in anti-inflammatory biomarkers such as Interleukin-10.

## Appendices

**Table 10:** Cochrane Risk of Bias Assessment Tool Detailed risk of bias assessment tool.

Study	Selection bias (Random sequence generation)	Selection bias (Allocation concealment)	Reporting bias (Selective reporting)	Other sources of bias	Performance bias (Blinding participants and personnel)	Detection bias (Blinding outcome assessment)	Attrition bias (Incomplete outcome data)	Final paper quality
Ballini et al. [17]	Unclear: The study does not provide enough detail on how participants were allocated to intervention groups, although it states this was a randomised, placebo-controlled trial and that 40 patients were randomly recruited. Therefore, the risk of bias remains unclear.	Low: Allocation was performed in a randomised manner (computer-based allocation).	Unclear: No comments regarding selective reporting or measures to address reporting bias.	Low: To avoid significant bias, patients with systemic disease affecting liver, kidney, and thyroid were excluded.	Low: This was a double-blind trial; participants in both groups received identical packaging.	Unclear: The trial is described as double-blind (patients and experimenters), but there is no mention of blinding of outcome assessors.	Low: A clear recruitment diagram with adequate information on exclusions is provided; all participants recruited are accounted for in the results and tables.	Fair
Bjarnason et al. [18]	Low: Participants were monitored during this stage completed in King’s College Hospital and the Department of Pharmacy at Guy’s & King’s College Hospital.	Unclear: Blinding of allocation to treatment group was carried out, but completed study packs were labelled and handed to patients by the manufacturer.	Unclear:No comments regarding selective reporting or measures to address reporting bias.	Low: No other bias detected.	Low: The placebo capsule was identical in appearance and taste and provided in identical packaging supplied by the manufacturer.	Unclear: Blinding of allocation to treatment group was carried out, but the completed study packs were labelled and handed over by the manufacturer.	Low: An intention-to-treat principle was used; no additional bias due to dropout.	Fair
Fan et al. [19]	Low: There were no differences between control and test groups with respect to sex, age, disease duration, or number of disease onsets.	Unclear: No mention of allocation concealment.	Unclear: No comments regarding selective reporting or measures to address reporting bias.	Low: No other bias detected.	High: No evidence of blinding of participants; control and test groups received different regimens.	Unclear: It is unclear whether outcome assessors were blinded.	Low: Complete data were available for all 40 participants initially enrolled.	Poor
Hegazy [20]	Low: Patients were randomly selected from Tanta University Hospital.	Unclear: Patients were allocated to probiotics or placebo, but the allocation method is unclear.	Low: No selective outcome reporting.	Low: No other bias detected.	Unclear: Although both groups had similar regimens, the treatment group received sachets whereas the placebo group did not, suggesting blinding of participants/personnel may not have been maintained.	Unclear: It is unclear whether outcome assessors were blinded; the paper states patients were blinded, but assessor blinding is not specified.	High: “This study might have biases that may affect the results since there are only 2 probiotics groups, and the number of patients is too small to provide sufficient information as to the extent of probiotic administration in UC patients.”	Poor
Huang et al. [21]	Low: Subjects were allocated using a random digit table method.	Low: Little information on allocation concealment; however, patients were not informed about their randomisation group, making bias unlikely.	Low: No evidence of selective outcome reporting.	Low: No other bias detected.	Low: Although blinding of participants/personnel is not explicitly mentioned, group 1 had an additional 2 months of placebo and group 2 had an additional 2 months of probiotics; therefore, patients and doctors could not have been blinded.	Unclear: Not enough information to assess detection bias.	Low: All patients present at baseline were accounted for in the results.	Good
Ishikawa et al. [22]	Low: Subjects drew lots, randomly assigning themselves to the group with bifidobacteria-fermented milk supplementation (BFM group) or without (control group).	Low: No explicit allocation concealment, but drawing lots meant allocation was unlikely to be influenced by researchers.	Low: No evidence of selective outcome reporting.	Low: No other biases detected.	High: No evidence of blinding of participants or personnel; groups received different treatment regimens.	Unclear: Assessor blinding is not reported; supplementation was administered manually, so assessors likely knew group assignment.	Low: No attrition bias; all enrolled patients were accounted for in the results.	Fair
Kato et al. [23]	Low: Twenty subjects were randomly assigned to test (n = 10; BFM supplementation) or control (n = 10; no BFM).	Low: Researchers did not know allocations; randomisation makes it unlikely subjects knew their group.	Low: No evidence of selective outcome reporting; all outcomes listed.	Low: No other bias detected.	Low: Double-blind; both participants and personnel were blinded. Treatment appearances were indistinguishable between groups.	Low: Histopathologists and physicians were blinded to treatment outcomes.	Low: The results section clearly accounts for all enrolled patients.	Good
Kruis et al. [24]	Low: Randomisation was carried out in a double-blind manner (allocation concealment not described).	Low: Randomisation performed; method of treatment allocation not detailed (computerised allocation to groups mentioned).	Low: All outcomes were reported; no evidence of selective reporting.	Low: No other bias detected.	Low: Double-blind, double-dummy randomised trial; both participants and personnel were blinded.	Low: Researchers and assessors were blinded.	Low: Clear flow chart for loss to follow-up; exclusions justified and accounted for. Intention-to-treat analysis minimised attrition bias.	Good
Li et al. [25]	Low: Subjects were divided into experimental and control groups according to a random digits table.	Low: The random digits table suggests a design with low risk of selection bias.	Low: No evidence of selective reporting.	Low: No other bias detected.	Unclear: No explicit statement on participant blinding; groups received different regimens, which may affect performance bias.	Low: To reduce experimental bias, different researchers handled clinical treatment, biochemical measurement, and microbiology.	Low: All participants were enrolled and accounted for in the results.	Fair
Wildt et al. [26]	Low: Participants were randomised using a sequence provided by a statistician not involved in the trial.	Unclear: A list with the allocation sequence was available to the pharmacist; concealment method not fully explained.	Low: No evidence of selective reporting.	Low: No other bias detected.	Low: Double-blind controlled trial; participants and personnel were unaware of group assignment, with the same regimens in both groups.	Low: The laboratory was blinded to the randomisation code.	Low: One patient was excluded on poor grounds; the remaining 20 were included in analysis and accounted for.	Good
Ng et al. [27]	Low: This was a double-blind study in which both participants and personnel were blinded.	Unclear: It is unclear whether the allocation sequence was randomised; allocation concealment was not mentioned.	Low: No evidence of selective outcome reporting.	Low: No other forms of bias detected.	Low: This was a double-blind study in which both participants and personnel were blinded.	Unclear: No clear mention of whether outcome assessors were blinded.	Low: All participants were enrolled and accounted for in the results; no additional attrition bias in analyses.	Fair
Yilmaz et al. [28]	Low: Eligible patients were selected randomly to receive treatment.	Low: There is no evidence of allocation concealment.	Low: No evidence of selective reporting.	Low: No other bias detected.	High: No evidence of blinding; placebo and active tablets differed, so participants were aware of group assignment and a control could not be prepared.	High: No evidence of assessor blinding, which could have led to high detection bias.	Low: All participants were accounted for and included in the results.	Poor
Cui et al. [29]	Unclear: Patients were randomly administered probiotic or placebo, but the randomisation method is not described.	Unclear: No description of allocation concealment procedures, raising risk of bias.	Low: Results appear consistent, but no trial registration or protocol is available to confirm selective reporting was avoided.	Low: Baseline characteristics appear balanced; no major confounders identified.	Low: A placebo (starch) capsule identical in appearance to the probiotic capsule was used, suggesting patients and treating clinicians were blinded.	Unclear: While blinding of patients and personnel is described, there is no clear statement about blinding of outcome assessors for endoscopic, histological, or molecular analyses.	Low: All 30 patients were accounted for, with relapses and outcomes clearly reported; no major loss to follow-up indicated.	Fair

**Table 11:** Table of results of the studies used in this review RCT - Randomised control trial; N - number of participants; M - Male; F - Female; CFU - Colony forming units; BAP - Biological Antioxidant Potential; d-ROM - derivatives of Reactive Oxygen Metabolites; CRP - C reactive protein; IL - Interleukins; SCFA - Short-Chain Fatty Acids; IgA - Immunoglobulin A; CD4 T cell - Cluster of Differentiation 4 T lymphocyte; CD8 T cell - Cluster of Differentiation 8 T lymphocyte; Colonic NF-κB p65 - Nuclear Factor kappa-light-chain-enhancer of activated B cells, p65 subunit; TNF-α - Tumor Necrosis Factor alpha; MPO - Myeloperoxidase; mRNA - messenger Ribonucleic Acid; UC - Ulcerative Colitis; IL-12p70 - Interleukin-12; p70 heterodimer, the biologically active form of IL-12 (composed of p35 + p40 subunits); IL-12p40 - Interleukin-12, p40 subunit (part of IL-12 and IL-23); TLR - Toll-Like Receptor; CD11 - Cluster of Differentiation; HLA - Human Leukocyte Antigen; RT-PCR - Reverse Transcription Polymerase Chain Reaction; EMSA - Electrophoretic Mobility Shift Assay; BIFICO - Bifidobacterium longum; Lactobacillus acidophilus; Enterococcus faecalis

Author and Year	Study design and cohort	Intervention	Biomarkers assessed	Summary of findings
Ballini et al., 2019 [17]	Randomised controlled trial (RCT), parallel study, placebo-controlled; conducted across 2 Italian hospitals, 1 Italian research institute, and 1 Albanian hospital. N = 40; Age: 30–60 (mean 45); Sex: 24 female / 16 male.	Tablet containing 15 probiotic strains (Hyperbiotic PRO-15), 75 × 10^9 CFU daily for 3 months. Placebo tablet.	Oxidative stress (measured by Biological Antioxidant Potential [BAP] and Derivatives of Reactive Oxygen Metabolites [d-ROM]).	d-ROM and BAP values decreased significantly in the probiotic group compared with the control group.
Bjarnason et al., 2019 [18]	RCT, parallel study, placebo-controlled; King’s College London. N = 81; Age: 18–70.	1 ml/kg/day of Symprove (10 billion live bacteria per 50 ml) for 4 weeks. Placebo given. Maintenance treatment included 5-aminosalicylic acid and azathioprine.	Faecal calprotectin, haemoglobin, haematocrit, white blood cell count, erythrocyte sedimentation rate (ESR), C-reactive protein (CRP).	Faecal calprotectin was markedly higher in placebo group vs probiotic group. CRP showed no significant difference. Four clinical relapses occurred, all in the placebo group, each with calprotectin > 250 μg/g.
Fan et al., 2019 [19]	RCT, parallel study, no placebo; Fujian University, China. N = 31; Mean age: probiotic group 42.56, control group 39.97; Sex: 20 male / 20 female.	Two tablets of Bifico three times daily for 40 days. Both groups also received 1–2 Pentasa.	α-1 antitrypsin, β2-microglobulin, high-sensitivity CRP, interleukin-6 (IL-6), interleukin-4 (IL-4), faecal lactoferrin.	α-1 antitrypsin, β2-microglobulin, high-sensitivity CRP, and IL-6 were significantly lower in the probiotic group compared with controls. IL-4 was significantly higher in the probiotic group. Faecal lactoferrin decreased significantly in the probiotic group. Baseline markers were similar in both groups.
Hegazy, 2018 [20]	RCT, parallel study, no placebo; Sanxia Central Hospital, China. N = 360; Mean age: 42.2; Sex: 90 female / 90 male.	Two Bifid triple viable capsules three times daily for 8 weeks.	Tumour necrosis factor-α (TNF-α), interleukin-8 (IL-8), interleukin-10 (IL-10).	Before treatment, no significant differences in TNF-α or IL-10 between groups. After treatment, TNF-α and IL-8 decreased significantly in both groups. IL-10 was significantly higher in the probiotic group compared with control.
Ishikawa, 2003 [21]	RCT, parallel study, no placebo; Tokyo, Japan. N = 21; Age: 39–60; Sex: 11 male / 10 female.	100 ml of bifidobacteria-fermented milk (10 billion bacteria) daily for 1 year.	Total protein, albumin, total cholesterol, triglycerides, faecal organic acids (succinate, lactate, formate, acetate, propionate, iso-butyrate, butyrate, iso-valerate, valerate).	No significant differences between groups, except butyrate, which decreased significantly in the probiotic group.
Kato et al., 2004 [22]	RCT, parallel study, placebo-controlled; Tokyo, Japan. N = 20; Mean age: 30; Sex: 10 male / 10 female.	100 ml/day bifidobacteria-fermented milk (10 billion bacteria). Placebo: Yakult without live bacteria. Duration: 12 weeks.	Faecal organic acids: succinate, lactic, acetic, propionic, butyric; total short-chain fatty acids (SCFA).	Propionic acid, butyric acid, and total SCFA increased significantly in the probiotic group compared with controls.
Kruis et al., 2004 [23]	RCT, parallel study, placebo-controlled; Germany. N = 175; Mean age: 42; Sex: probiotic group 56.8% male, control 52.7% male.	Mutaflor: 100 mg days 1–4, 200 mg thereafter up to 12 months. Placebo tablet.	ESR, CRP, orosomucoid, blood count, liver enzymes, creatinine, serum iron, serum albumin.	No biomarker data reported.
Li et al., 2012 [24]	RCT, parallel study, no placebo; Nanchang University, China. N = 82; Age: 21–70; Sex: 47 male / 37 female.	Two Bifid triple viable capsules three times daily. Both groups also received 5-ASA.	Blood: peripheral immunoglobulins, complement C3 and C4, T cell subgroups, ESR. Immunohistochemistry: IL-1β, IL-10, immunoglobulin A (IgA), CD4 T cells, CD8 T cells.	No significant differences in blood immunoglobulins, complement, or T cell subgroups. CD4:CD8 ratio increased significantly in the probiotic group. IgA and IL-10 increased significantly in colonic mucosa, IL-1β decreased significantly.
Wildt et al., 2011 [25]	RCT, parallel study, placebo-controlled; Denmark (2 centres). N = 22; Mean age: 38; Sex: 10 male / 32 female.	Two capsules of Probio-Tec AB-25 three times daily (2.5 × 10^10 CFU per capsule) for 52 weeks. Placebo capsule.	Haemoglobin, CRP, white blood cell count, albumin.	No significant differences between groups.
Huang et al., 2010 [26]	RCT, parallel study, placebo-controlled; Egypt. N = 40; Age: 46; Sex: 30 male / 14 female.	10 billion bacteria (Lactobacillus delbrueckii and Lactobacillus fermentum) daily for 8 weeks. Both groups also received sulfasalazine. Placebo: starch. Healthy control group included.	Colonic myeloperoxidase (MPO), IL-6, nuclear factor κB p65 (NF-κB p65, immunohistochemistry), TNF-α (mRNA), faecal calprotectin.	MPO decreased significantly in both sulfasalazine and probiotic groups. IL-6 decreased significantly after treatment. Calprotectin decreased significantly in probiotic and sulfasalazine groups. NF-κB p65 expression decreased significantly in probiotic group compared with others. TNF-α mRNA decreased significantly after treatment with probiotics and sulfasalazine.
Yilmaz et al., 2019 [27]	RCT, parallel study, no placebo; Turkey. N = 25; Age: 19–76; Sex: 13 male / 12 female.	400 ml of kefir twice daily for 4 weeks.	Haemoglobin, CRP, ESR.	No significant differences observed.
Ng et al., 2010 [28]	RCT, parallel study, no placebo; London. N = 28; Age: 21–70; Sex: 11 male / 17 female.	Two sachets of VSL#3 daily (3600 billion bacteria per sachet) for 8 weeks. Comparator: prednisolone 40 mg daily. Placebo: starch.	Colonic dendritic cells (HLA-DR+ Lin–, CD11+ and CD11–). Cytokines (IL-10, IL-12p40, IL-6, IL-13, IL-4, IL-5, IL-1β, TNF-α, IL-12p70) and receptor expression (CD40, CD86, toll-like receptors [TLR]-2, TLR-4).	Dendritic cells significantly increased in UC patients. IL-10 increased in probiotic responders. IL-12p70 decreased. No significant change in IL-6 or IL-13. TLR-2 decreased significantly with probiotics; no changes in CD40, CD86, or TLR-4. Non-responders showed smaller IL-12p40 reduction and minimal IL-10 increase.
Cui et al., 2004 [29]	RCT, parallel study, placebo-controlled; PLA Institute, China. N = 60.	1.26 g/day Bifid Triple Viable for 8 weeks. Placebo: starch. Both groups also received sulfasalazine and glucocorticoids.	NF-κB (p65), inhibitor of NF-κB (IκB), DNA-binding activity of NF-κB (electrophoretic mobility shift assay, EMSA), cytokines by RT-PCR: IL-1, TNF-α, IL-10, IL-1β.	NF-κB expression decreased significantly in the probiotic group. IκB expression decreased. EMSA showed higher NF-κB activation in patients not taking probiotics. TNF-α and IL-1 decreased significantly, IL-10 increased significantly with probiotics. Placebo group showed no significant changes.
