# Correction: Biomarkers of Probiotic Therapy in Ulcerative Colitis: A Systematic Review of Mechanisms Underlying Remission

**DOI:** 10.7759/cureus.c412

**Published:** 2026-03-10

**Authors:** Mohammad E Farhad, Ibrahim Belal, Mohamed Baana, Caroline Childs, Moussa Al-Rufayie

**Affiliations:** 1 General Surgery, Hillingdon Hospital, London, GBR; 2 General Medicine, Royal London Hospital, London, GBR; 3 Emergency Medicine, Hillingdon Hospital, London, GBR; 4 Human Development and Health, University of Southampton, London, GBR; 5 Emergency Medicine, London North West University Healthcare NHS Trust, London, GBR

Several studies cited in this article assessed a probiotic formulation previously known as VSL#3. The probiotic formulation that was assessed in these studies is now known by the generic name 'De Simone Formulation'. The current product known as VSL#3 is not the same formulation as the De Simone Formulation. The De Simone Formulation is available under the brand names Visbiome (USA) and Vivomixx (Europe).

